# All‐Optical Dual‐Angle Diffractive Neural Networks for 2D Direction‐of‐Arrival Estimation

**DOI:** 10.1002/nap2.70315

**Published:** 2026-10-22

**Authors:** Chenjie Xiong, Shuo Wang, Jianzhou Huang, Jia Zhang, Luosha Zhang, Yu Wang, Jin Tao, Chengjun Zou, Bin Hu

**Affiliations:** ^1^ School of Optics and Photonics Beijing Institute of Technology Beijing China; ^2^ National Key Laboratory on Near‐surface Detection Beijing Institute of Technology Beijing China; ^3^ Institute of Microelectronics Chinese Academy of Sciences Beijing China; ^4^ University of Chinese Academy of Sciences Beijing China; ^5^ China Academy of Aerospace Science and Innovation Beijing China; ^6^ Institute of Applied Physics Abbe Center of Photonics Friedrich Schiller University Jena Jena Germany; ^7^ State Key Laboratory of Optical Communication Technologies and Networks China Information Communication Technologies Group Corporation Wuhan China

**Keywords:** diffractive neural networks, direction‐of‐arrival estimation, terahertz communications

## Abstract

Accurate direction‐of‐arrival (DoA) estimation in real time is essential for stable terahertz (THz) communications. To overcome the limitations of previous diffractive methods that are restricted to 1D sensing or depend on rotation, and to advance their practical deployment, we propose and experimentally validate an all‐optical dual‐angle diffractive neural network (DA‐DNN) capable of simultaneous 2D angular classification. At the heart of our design is a diffractive aperture placed before the neural network as a physical encoder. It converts the phase‐only angular information carried by the incident wave into a complex optical field enriched with amplitude features, thereby producing robust spatial encoding sensitive to angle. The system is modeled using the Rayleigh–Sommerfeld diffraction integral and optimized through end‐to‐end backpropagation. We fabricate a compact, monolithically integrated DA‐DNN device using 3D printing at high resolution and evaluate it through full‐wave simulations and THz experiments that emulate communication environments. The system accurately recognizes 64 angular combinations over a $\left.\left[-40{}^{\circ},40{}^{\circ}\right.\right)$ range, providing a passive and scalable optical solution with low latency for THz communications.

## Graphical abstract

A diffractive neural network with an integrated spatial encoder resolves the direction of an incoming terahertz signal entirely through optical propagation. After the signal passes through the cross‐shaped input and two 3D‐printed diffractive layers, two detector arrays read its two incident angles simultaneously. The compact system therefore performs direct dual‐angle sensing without electronic image processing.

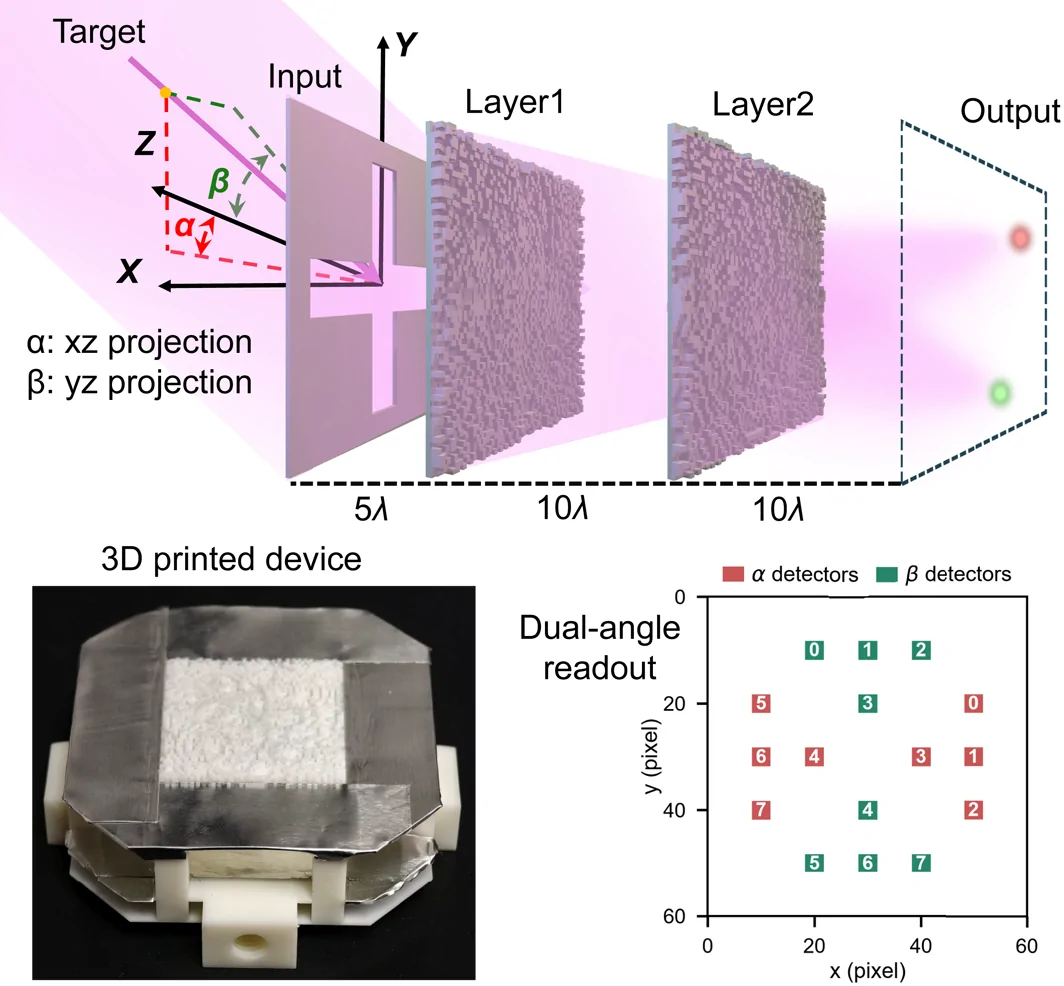

## 1 Introduction

With the rapid development of wireless communication technologies, direction‐of‐arrival (DoA) estimation has become a core enabling technology for numerous advanced applications [1, 2, 3, 4, 5, 6]. In remote sensing, it supports precise target localization and environmental reconstruction; in modern communication systems, it is fundamental to beamforming optimization and interference mitigation; and in surveillance systems, it enables target tracking and situational awareness [7, 8, 9, 10, 11, 12]. Collaborative systems involving multiple unmanned aerial vehicles (UAVs) also rely heavily on fast and reliable DoA estimation with low latency for millimeter‐wave and terahertz (THz) links, radar localization, and autonomous navigation [13, 14].

Conventional DoA estimation approaches, whether optical methods such as lenses or electronic methods such as multiple signal classification (MUSIC) and estimation of signal parameters via rotational invariance techniques (ESPRIT), have traditionally focused on achieving high angular resolution. Both approaches, however, encounter fundamental trade‐offs. Electronic methods require antenna arrays, sophisticated radio‐frequency front ends, and digital signal processing with high power demand, often involving computationally expensive matrix operations and spectral searches that introduce substantial latency and energy consumption [15, 16, 17]. Optical lenses, although apparently simple, critically depend on ideal conditions such as collimated plane‐wave illumination and require additional calibration and computationally intensive electronic postprocessing to retrieve focal spot positions. Under realistic conditions involving oblique incidence, distorted wavefronts, or large incident angles, lenses often produce disordered speckle patterns and large, abrupt focal spot displacements, thereby obscuring directional information and lowering sensing accuracy.

Recently, diffractive neural networks (DNNs) have attracted considerable interest because they operate passively in real time and with high energy efficiency [18]. DNNs and related learning‐assisted photonic systems have been successfully applied to image classification [19, 20, 21, 22, 23, 24], optical field modulation and encryption [25, 26, 27, 28, 29, 30, 31], computational imaging [32, 33, 34, 35, 36, 37, 38, 39, 40], spatial signal recognition [41, 42], and information processing [43, 44]. Metasurface and flat‐optics devices have likewise enabled reconfigurable filtering, scattering that depends on direction, and THz information processing [45, 46, 47, 48]. In DoA estimation, DNNs use trainable diffractive layers to map incident electromagnetic waves directly into spatial intensity patterns. This optical mapping is robust against wavefront distortions and can support a large angular sensing range, or field of view (FOV). Diffractive neural networks have also been used for joint estimation of the elevation angle and frequency of incident electromagnetic waves [49]. Gao et al. [50] recently demonstrated a millimeter‐wave super‐resolution diffractive neural network (S‐DNN) that achieved angular estimation beyond the diffraction limit, highlighting the potential of diffractive systems for enhanced angular discrimination. Despite this super‐resolution capability, the reported system was limited to 1D DoA sensing and required mechanical rotation of the S‐DNN device for 2D angular characterization. Multiple devices or rotating mechanisms can extend the sensing dimensionality, but they inevitably increase system complexity, physical size, and downstream electronic processing latency. Direct and simultaneous decoding of 2D angular information from an incident wavefront using a single passive diffractive network therefore remains a key challenge for all‐optical DoA estimation.

To overcome these challenges, we propose and experimentally demonstrate an all‐optical dual‐angle diffractive neural network for DoA estimation. We found that directly extending the architecture reported by Gao et al. [50] was insufficient for accurate 2D DoA sensing. Relying only on the phase information of the incident wavefront makes the estimation process sensitive to free‐space scattering and random structural imperfections in the diffractive layers, leading to unstable training and reduced accuracy. Simply increasing the number of diffractive layers also lowers the signal‐to‐noise ratio and narrows the sensing FOV because waves incident at large angles cannot propagate efficiently through all layers.

To address these limitations, we introduce an aperture before the dual‐angle diffractive neural network (DA‐DNN) as a spatial encoding layer. The encoder applies a prescribed amplitude modulation to the incident wavefront and produces a distinct spatial complex field for each 2D angular state. This transformation introduces features of higher dimensionality and strengthens the one‐to‐one correspondence between incident direction and the field distribution at the DA‐DNN input plane. The subsequent diffractive layers collectively act as a spatial decoder and angular classifier. This all‐optical encoder–decoder architecture enhances 2D angular sensing without the additional hardware complexity of previous approaches based on rotation [50].

The DA‐DNN is modeled using the Rayleigh–Sommerfeld diffraction integral and optimized through a customized end‐to‐end backpropagation algorithm. We fabricate a compact prototype using 3D printing at high resolution and evaluate it through full‐wave simulations and THz experiments that emulate practical communication scenarios. The experimental results show that the DA‐DNN accurately classifies 64 angular combinations across a wide $\left.\left[-40{}^{\circ},40{}^{\circ}\right.\right)$ FOV, providing a practical route toward passive, compact, and intelligent all‐optical front ends. The proposed method is particularly suited to 3D communication systems requiring operation in real time and surveillance requiring high precision on platforms with limited resources.

## 2 Results and Discussion

### 2.1 Design and Training of DA‐DNNs With Spatial Encoders

The working principle of the DA‐DNN is to project the directional information of a target source arriving from an arbitrary direction onto two orthogonal angular components, as shown in Figure 1a. These components are independently classified into their corresponding angular intervals, thereby determining the target direction relative to the DA‐DNN. In conventional DoA estimation, the source direction is typically parameterized by the zenith angle θ (0°–90°) and the azimuth angle φ (0°–360°). Although an ideal deep neural network with sufficient complexity could learn arbitrary transformations, the (θ,φ) parameterization substantially increases the learning complexity of a shallow diffractive network because of its unequal angular ranges and inherent nonlinear coupling. To alleviate this analog computational burden, we adopt an alternative coordinate system that simplifies the mapping between incident direction and output classification.

**FIGURE 1:**
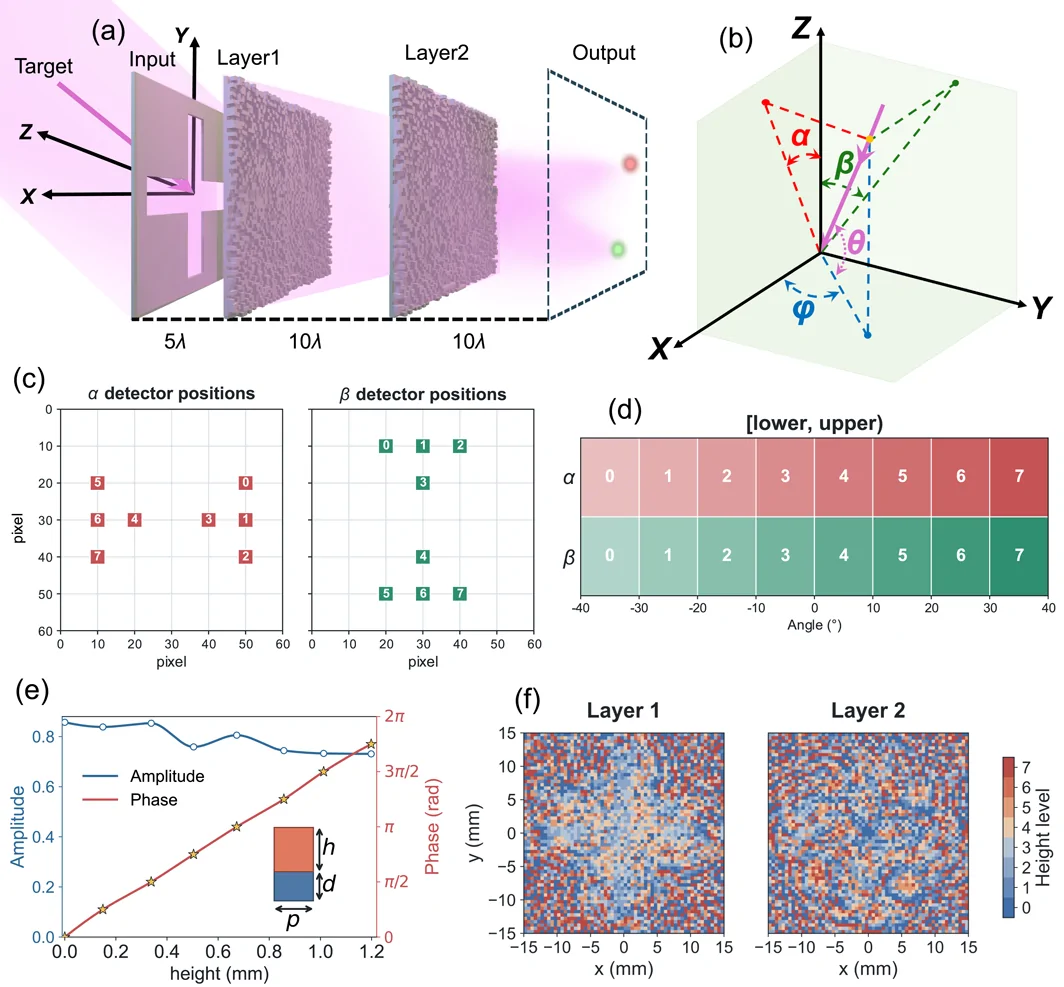
System architecture, angular definitions, and output encoding of the dual‐angle DA‐DNN. (a) The incident field from a target passes successively through the rectangular cross input aperture, DOE1, and DOE2 before reaching the output detection plane. The propagation distances from the aperture to the first layer, between the two layers, and from the second layer to the output plane are indicated. (b) Coordinate definitions of the zenith angle θ, azimuth angle φ, and the signed projected angles α and β used in this work. The angle α is defined in the xz projection plane and β in the yz projection plane. (c) Two disjoint detector groups on the output plane. Red regions read the α class, and green regions read the β class; the numbers denote detector indices. (d) Class partitioning of α and β over $\left.\left[-40{}^{\circ},40{}^{\circ}\right.\right)$. Each component contains eight left‐closed, right‐open 10° intervals indexed from 0 to 7. (e) Reference transmission amplitudes and phases of the eight discrete height levels, with a unit‐cell period p=0.5 mm, base thickness d=0.5 mm, and variable height h. (f) Height distributions of the two optimized DOEs, where color denotes the discrete height level.

Specifically, we introduce a new pair of angular parameters, (α,β), to encode the target direction. This coordinate system provides a more balanced and direct representation. As illustrated in Figure 1b, the target direction is decomposed into a horizontal angle α (red dashed line) and a vertical angle β (green dashed line). This representation allows the network to focus on angular classification and achieve higher accuracy with substantially reduced complexity. The precise definitions of all four angles are provided in Supporting Information S1: Section S1, and the conversion relations between (θ,φ) and (α,β) are

(1) $$\left\{\begin{array}{cc} \theta & =\tan^{-1}\left(\sqrt{{\left(\tan\;\alpha \right)}^{2}+{\left(\tan\;\beta \right)}^{2}}\right),\\ \varphi & =\text{atan}2\;\left(\tan\;\beta ,\tan\;\alpha \right). \end{array}\right.$$

At the network input, we introduce a diffractive aperture that encodes phase‐only DoA information into a structured spatial intensity pattern.

For simultaneous dual‐angle recognition, the training and test datasets consist of far‐field plane waves corresponding to targets at different incident angles. The incident field is modeled as [51]

(2) $$E\left(x,y\right)=\exp\left[jk\left(x\;\sin\;\theta \;\cos\;\varphi +y\;\sin\;\theta \;\sin\;\varphi \right)\right],$$

The FOV is set to 80° during training, with $\alpha ,\beta \in \left.\left[-40{}^{\circ},40{}^{\circ}\right.\right)$. Both the horizontal and vertical angles are discretized into eight intervals with a width of 10°, corresponding to angular intervals $\alpha_{j}$ and $\beta_{k}$ for j,k=0,…,7. Each interval includes its left boundary and excludes its right boundary. The resulting 16 angular intervals are mapped to horizontal (red) and vertical (green) detectors indexed from 0 to 7, respectively, as shown in Figure 1c. The mapping between detector indices and angular intervals is shown in Figure 1d; detectors with the same index in the horizontal and vertical directions correspond to the same angular range. For each pair of intervals $\alpha_{j}$ and $\beta_{k}$, all integer angles within the intervals are sampled and combined, and Equation (2) is used to generate the corresponding phase distributions. Because both α and β take integer values over $\left.\left[-40{}^{\circ},40{}^{\circ}\right.\right)$, each direction contains 80 discrete angles, resulting in a training set of 80×80=6,400 samples. The test set is generated using the same procedure, except that the selected angles lie strictly within the central region of each 10° interval to avoid boundary effects. For this purpose, the outer 1/20 portion at each end of every interval is removed, and the refined intervals are sampled at an angular step of 0.1° to construct the test set.

As illustrated in Figure 1a, the DA‐DNN comprises three main components: an aperture layer, two diffractive hidden layers (composed of diffractive optical elements (DOEs)), and an output detection plane. We intentionally restrict the network to two layers to preserve sensing capability over a large FOV. The incident field first passes through the aperture and is spatially filtered. The complex amplitude immediately after the aperture is

(3) $$E_{\text{in}}=M⊙E,$$

where E is the discrete incident complex field, M is the aperture amplitude transmission mask, and ⊙ denotes element‐wise multiplication.

The transmitted field then propagates a distance of 5λ to the first hidden layer. This propagation is modeled using the Rayleigh–Sommerfeld diffraction integral [52], yielding

(4) $$E_{\text{in}}^{1}=D_{1}E_{\text{in}},$$

where $D_{1}$ is a complex symmetric matrix representing diffraction from the aperture to the front surface of the first hidden layer [53].

Each hidden layer contains a 60×60 array of diffractive elements. Under oblique incidence, the electromagnetic response of each element depends on its height, the incident field, and the electromagnetic environment formed by adjacent elements. Treating each element independently therefore does not fully describe the changes in amplitude and phase caused by oblique incidence and local electromagnetic coupling. Following the response modeling concept of the knowledge‐bridged neural network (KBNN) introduced by Ma et al. [54], we use a Height‐Conditioned Complex‐Field Response Network (HCFR‐Net) to calculate how the complex vector field changes as it passes through each DOE layer. Each DOE height map contains eight discrete height levels. Their reference transmission amplitudes and phases under normal incidence are shown in Figure 1e. The field transformation through layer l is expressed as

(5) $$E_{l}^{\text{out}}=HCFR‐Net\left(E_{l}^{\text{in}},H_{l}\right),\;l=1,2,$$

where $E_{l}^{\text{in}}$ is the incident complex vector field of layer l and contains the $E_{x}$, $E_{y}$, and $E_{z}$ components, $H_{l}$ is its eight‐level quantized height map, and $E_{l}^{\text{out}}$ is the transmitted complex vector field immediately after the layer. HCFR‐Net was trained separately using paired incident and transmitted complex‐field data generated by finite‐difference time‐domain (FDTD) simulations. Once this training was completed, its parameters were kept unchanged during the optimization of the DA‐DNN. The same trained HCFR‐Net was used for both DOE layers. HCFR‐Net calculated the field transformation through each DOE, whereas propagation between finite planes was calculated using the Rayleigh–Sommerfeld diffraction integral. Further information on the FDTD response dataset and its angular coverage is provided in Supporting Information S1: Section S2.

The output field of DOE1 propagates to DOE2, and the field modulated by DOE2 then propagates to the detection plane:

(6) $$E_{2}^{\text{in}}=D_{2}E_{1}^{\text{out}},$$

(7) $$Y=D_{2}E_{2}^{\text{out}},$$

The operator $D_{2}$ describes diffraction over the 10λ separation between the two hidden layers and also describes diffraction over the 10λ from the second hidden layer to the detection plane. The output intensity is $I=|Y{|}^{2}$.

Diffractive optical neural networks generally construct classification objectives from the optical signals collected by class detectors and optimize the diffractive layer parameters through error backpropagation. Following the hard‐sample emphasis of focal loss [55], we construct a joint dual‐angle objective comprising the negative log‐likelihoods of the two detector arrays, an energy margin penalty in the logarithmic domain, and adaptive sample weights. The loss uses the normalized detector energies in each array as class probabilities and adjusts each sample contribution according to the weaker classification margin of the two arrays and its current misclassification state:

(8) $$L_{B}=\frac{1}{B}\sum_{i=1}^{B}{\tilde{w}}_{i}\left[-\sum_{h\in \left\{\alpha ,\beta \right\}}\log p_{h,y_{h}^{\left(i\right)}}^{\left(i\right)}+\lambda \sum_{h\in \left\{\alpha ,\beta \right\}}{\left[\gamma -m_{h}^{\left(i\right)}\right]}_{+}\right].$$

Here, B is the minibatch size, corresponding to the number of incident‐angle pairs (α,β) processed in each optimization step, which is 128 in our work. h∈{α,β} denotes the two detector arrays, $p_{h,k}^{\left(i\right)}$ is the normalized energy in detector region k of array h for sample i, and $y_{h}^{\left(i\right)}$ is the true class for that detector array. The energy margin in the logarithmic domain between the true class and the strongest incorrect class is

(9) $$m_{h}^{\left(i\right)}=\log p_{h,y_{h}^{\left(i\right)}}^{\left(i\right)}-\max_{k\neq y_{h}^{\left(i\right)}}\;\log p_{h,k}^{\left(i\right)}.$$

The normalized detector energies used in Equations (8) and (9) are calculated separately within each detector array as

(10) $$p_{h,k}^{\left(i\right)}=\frac{E_{h,k}^{\left(i\right)}+\varepsilon_{\text{num}}}{\sum_{j=0}^{7}E_{h,j}^{\left(i\right)}+8\varepsilon_{\text{num}}},\;h\in \left\{\alpha ,\beta \right\}.$$

Here, $E_{h,k}^{\left(i\right)}$ is the energy collected by detector region k of array h for sample i. The numerical constant $\varepsilon_{\text{num}}=1.0\times 10^{-10}$ prevents zero probabilities and undefined logarithms.

The hinge term is defined as ${\left[\gamma -m_{h}^{\left(i\right)}\right]}_{+}=\max\left(\gamma -m_{h}^{\left(i\right)},0\right)$, with a target margin of γ=0.75 and a margin coefficient of λ=0.42. The margin penalty becomes zero when the normalized energy of the true‐class detector is at least exp(0.75)≈2.12 times that of the strongest competing detector. The coefficient λ controls the contribution of the margin penalty relative to the dual‐angle negative log‐likelihood.

The adaptive weight ${\tilde{w}}_{i}$ is determined by the weaker classification margin of the two detector arrays and the current misclassification state. The weights are normalized to have a mean of one within each minibatch, increasing the contribution of difficult samples without changing the overall loss scale.

To evaluate the effect of aperture geometry on dual‐angle classification, we compared hexagonal, diamond, six‐point star, square, triangular, circular, cross, and no aperture configurations under otherwise identical conditions. The aperture masks and the ablation results over the 80° angular range are shown in Figure 2. Figure 2a presents the masks, where yellow denotes an open region and the dark regions denote blocked regions. In Figure 2b, the α and β accuracies are the independent accuracies of the two angular components, whereas the joint accuracy requires both components to be correct. Additional aperture ablations over 40°, 16°, and 8° ranges, corresponding to class widths of 5°, 2°, and 1°, respectively, are provided in Supporting Information S1: Section S3.

**FIGURE 2:**
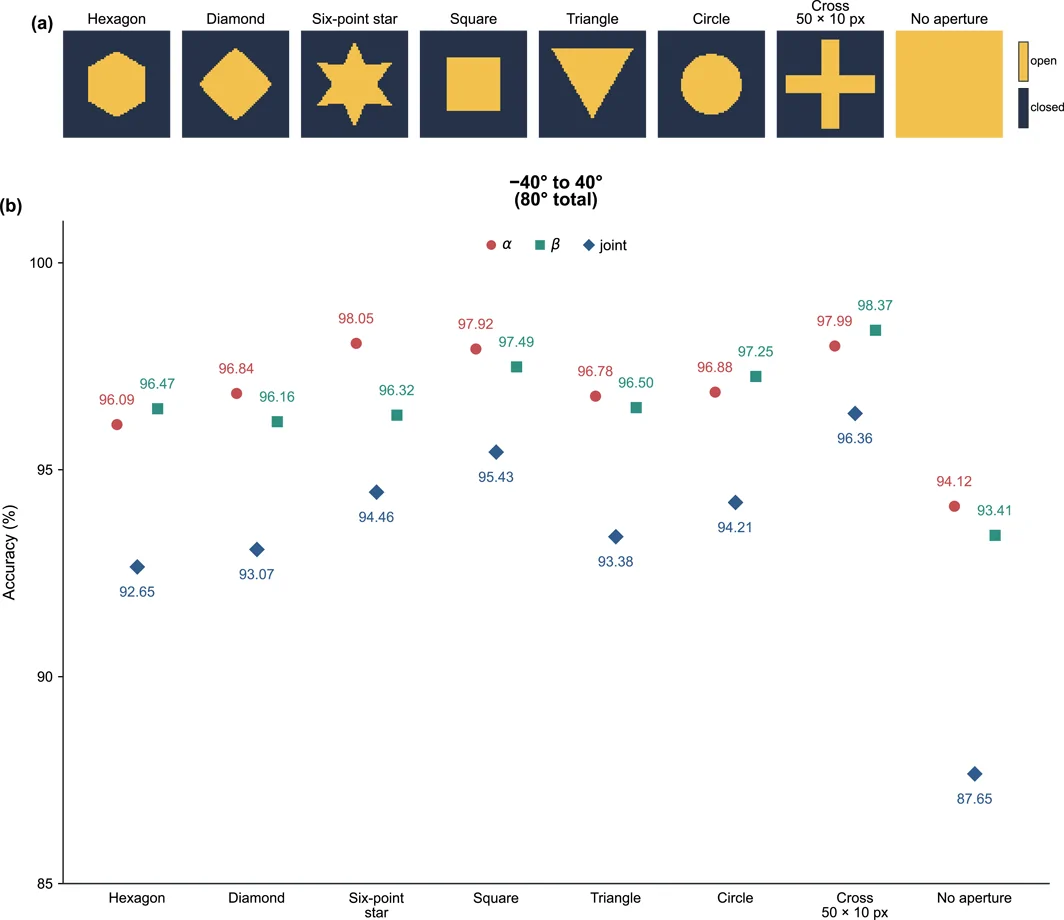
Effect of aperture geometry on dual‐angle classification over an 80° angular range. (a) The eight configurations, from left to right, are hexagonal, diamond, six‐point star, square, triangular, circular, cross, and no aperture. Yellow denotes an open region, and the dark regions denote blocked regions. (b) Independent α accuracy, independent β accuracy, and joint accuracy obtained under the same training conditions.

Over the 80° range, the cross aperture achieved a joint accuracy of 96.36%, compared with 87.65% without an aperture; the other six aperture shapes achieved 92.65%–95.43%. When the angular range was reduced to 40°, the cross‐ and no‐aperture configurations achieved joint accuracies of 99.17% and 98.72%, respectively. The corresponding values were 97.80% and 99.66% over the 16° range and 96.60% and 98.95% over the 8° range. The improvement provided by the cross aperture over direct input is therefore most pronounced across the 80° angular range.

After identifying the cross geometry, we further optimized the aperture dimensions. The detailed size ablation is presented in Supporting Information S1: Section S4. After careful consideration, we selected a 50×10 pixel cross aperture for subsequent experimental fabrication. The optimized height maps obtained with this 50×10 pixel cross aperture are shown in Figure 1f, and the DOEs were fabricated using 3D printing at high resolution.

Following this procedure, we implemented a DA‐DNN with two hidden layers for simultaneous recognition of the horizontal and vertical angles. The choice of two layers reflects a key physical constraint. An architecture with one layer did not converge in our tests, whereas architectures with two or more layers converged reliably. Because the FLGPWH04 resin used for 3D printing exhibits appreciable loss in the THz band, additional layers would reduce optical throughput and lower the detector signal‐to‐noise ratio. Under the present material constraint, two layers are therefore the optimal choice for balancing performance and implementability. The 60×60 resolution was selected according to the available computational resources, and the results consistently show that it is sufficient for accurate 2D DoA classification, illustrating the efficiency of the encoder–decoder architecture.

### 2.2 Device Fabrication and Experimental Results

Experimental validation required a DA‐DNN device with precise interlayer alignment, high mechanical strength, and stable geometry. We therefore adopted a monolithic fabrication strategy and printed the complete DA‐DNN in a single process using a Formlabs Form 3 3D printer at high resolution. The diffractive layers and the input diffractive encoder were fabricated from FLGPWH04 resin whose complex permittivity at 0.3 THz was measured as ε=2.919+0.085j using terahertz time‐domain spectroscopy (THz‐TDS) [56, 57, 58]. The diffractive layers were integrated into enlarged 5cm×5cm substrates and connected by eight peripheral vertical support columns. The columns had a 0.5cm×0.5cm cross section and heights of 0.5 and 1 cm, corresponding to the designed propagation distances. The insets in Figure 3a show the front and rear surfaces of the printed sample. The front incident surface surrounding the diffractive aperture was covered with aluminum foil tape to suppress stray incident radiation. This monolithic design, fabricated by 3D printing, provides high fabrication precision and substantially reduces the alignment difficulties commonly encountered in DNN devices.

**FIGURE 3:**
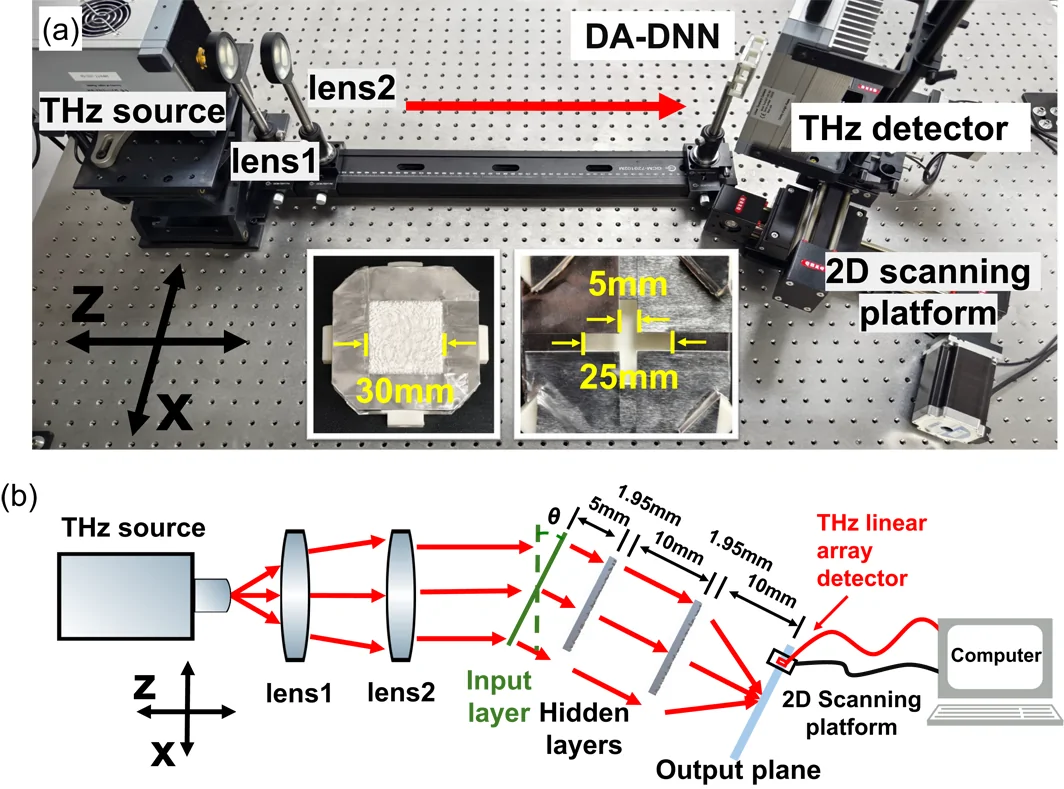
Experimental configuration. (a) Photograph of the complete DA‐DNN setup; the insets show the aperture side of the fabricated sample from the front and rear. (b) Schematic representation of the setup, including the THz emission module, DA‐DNN, and scanning detection module. For equivalent 2D angular measurements, the DA‐DNN and scanning detector are jointly rotated by θ about the center of the diffractive aperture, followed by a rotation of the DA‐DNN by φ about the device axis.

To characterize the sample performance, we constructed the experimental setup shown in Figure 3. Figure 3a,b presents the photograph and schematic, respectively. A 0.3‐THz continuous‐wave emitter (TeraSense IMPATT series) was used as the coherent source. Two TPX lenses (Tydex) collimated and expanded the THz wave, producing a uniform and clearly defined plane wavefront across the effective DA‐DNN aperture. After transmission through the DA‐DNN, a fast linear array detector (TeraSense TeraFAST series) measured the spatial distribution of the transmitted signal through 2D scanning. The recorded intensity data were then analyzed to obtain the angular detection results.

Because the source could not be translated in our experimental setup, we developed an equivalent 2D angular measurement method based on physical rotation of the DA‐DNN. The source remained fixed, whereas the device and detector were rotated together. As shown in Figure 3b, the DA‐DNN and scanning detection module were first rotated synchronously by θ about the center of the diffractive aperture. The device was then rotated by φ about its central axis to obtain equivalent measurements of α and β. The sample substrate was redesigned with multiple mounting slots to support different orientations; photographs of the redesigned device and its connection to the base are provided in Supporting Information S1: Section S5. Four azimuthal rotation states of 45°, 135°, 225°, and 315° were selected, as shown in Figure 4. Figure 4a presents the four physical rotation states and the corresponding change from the original x,y axes to the rotated u,v axes. The sample and detector plane were rotated together in every case. Figure 4b shows the equivalent optical path and the corresponding (θ,φ) angles after rotation. In this equivalent representation with the device fixed, clockwise rotation of the DA‐DNN preserves θ and produces the corresponding rotation of φ. For example, rotating the downstream optical path by θ=5.23° and the DA‐DNN clockwise by 45° gives the equivalent angles θ=5.23° and φ=−45°. Equation (1) then gives (α,β)=(3.7°,−3.7°).

**FIGURE 4:**
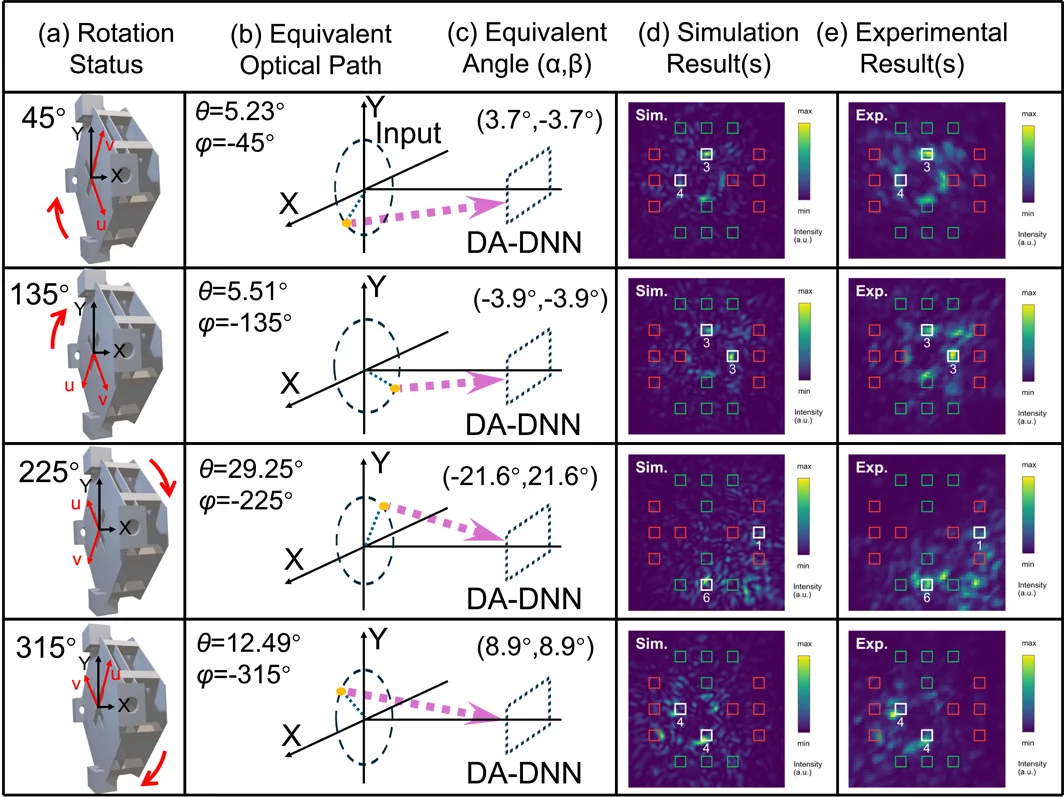
Equivalent dual‐angle experimental validation obtained by rotating the sample. The four rows correspond to clockwise sample rotations of 45°, 135°, 225°, and 315°. (a) Rotation angle and physical orientation of the sample. (b) Equivalent (θ,φ) angles and incident optical path after transforming the rotated sample into the fixed device coordinate system. (c) Corresponding projected angles (α,β) of (3.7°,−3.7°), (−3.9°,−3.9°), (−21.6°,21.6°), and (8.9°,8.9°). (d) FDTD full‐wave simulated output intensity distributions for the four equivalent angular states. (e) Experimental measurements under the same conditions. Red and green boxes denote the α and β detector regions, respectively; white boxes and numbers identify the target class in each detector group.

We performed a comprehensive validation using this method based on equivalent angles. Each row of Figure 4 corresponds to a different physical rotation angle, namely, 45°, 135°, 225°, or 315°, and successively presents the physical sample rotation, equivalent optical path, resulting 2D incident angles (α,β), full‐wave simulated output, and experimental measurement. Red and green boxes denote the predefined detector regions for horizontal and vertical angle recognition, whereas the white boxes identify the maximum in each detector array. Direct comparison of the simulated and experimental columns shows a high degree of agreement for every row. This agreement provides strong experimental evidence for the robustness and reliability of the DA‐DNN at arbitrary 2D incident angles and validates the scientific correctness of the equivalent measurement strategy. All tested angular configurations exhibit high classification accuracy, further demonstrating the effectiveness of the design.

To illustrate the angular sensing performance, we further examined the energy distributions across the 16 detectors using (α,β)=(−3.9°,−3.9°) as a representative incident state. Figure 5a–c presents the output intensity distributions obtained by Rayleigh–Sommerfeld calculation, FDTD full‐wave simulation, and experimental measurement; the corresponding measurement is also provided in Video S1. All three methods show that Detector 3 in both the horizontal and vertical arrays receives the maximum intensity, in close agreement with the true target direction and the mapping in Figure 1c. Figure 5d–f presents the calculated, simulated, and experimental intensity distributions over the horizontal and vertical detector arrays corresponding to the three intensity maps in Figure 5a–c. All distributions exhibit pronounced dominant peaks at Detector 3 in both arrays. These results validate the optical model and demonstrate that the fabricated DA‐DNN can reliably perform its intended angular discrimination. Beyond this representative case, we also conducted simulations and experimental validations for multiple angular combinations, as summarized in Supporting Information S1: Sections S6 and S7. These measurements further demonstrate the universal and robust angular recognition capability of the device across the designed $\left.\left[-40{}^{\circ},40{}^{\circ}\right.\right)$ wide FOV.

**FIGURE 5:**
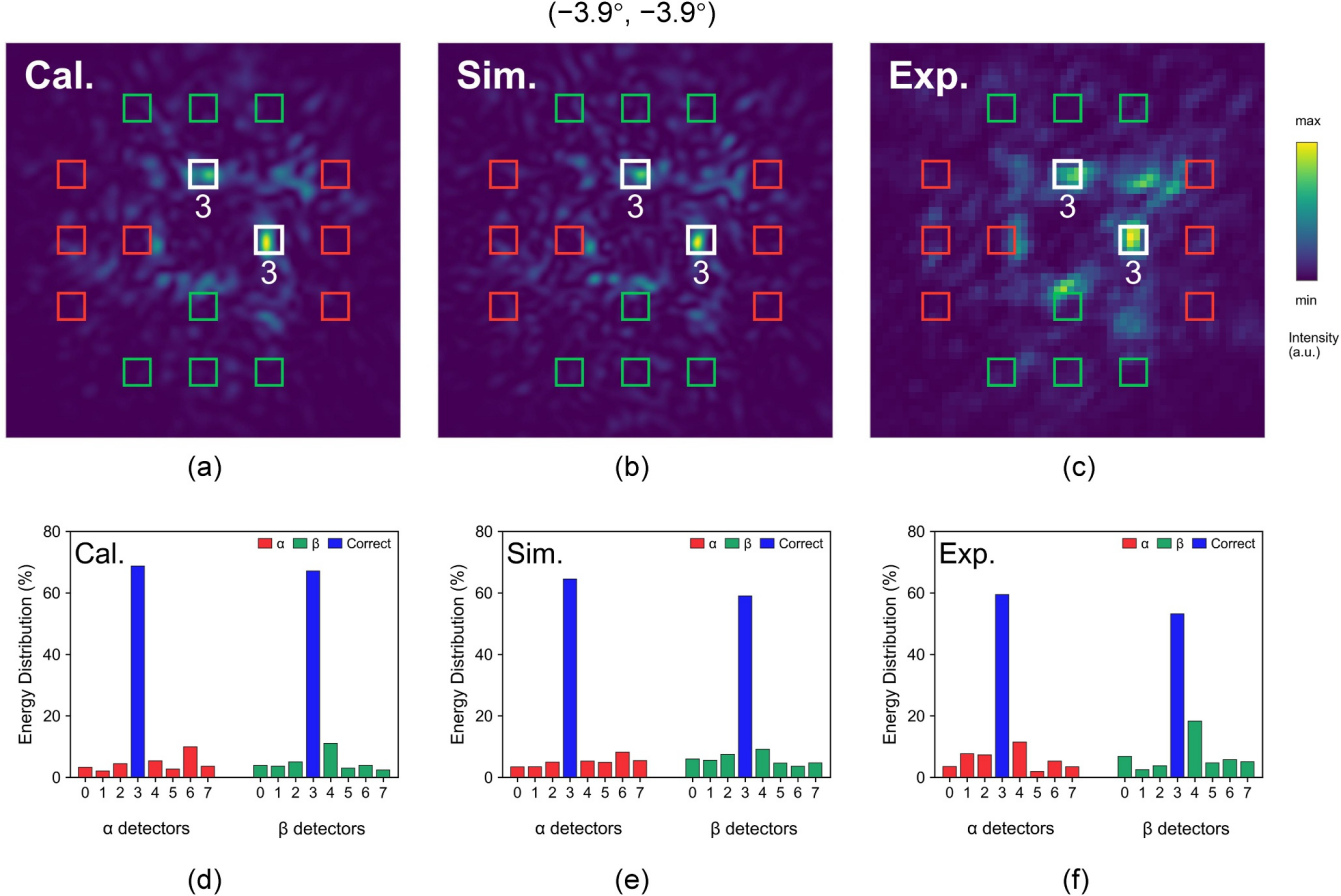
Dual‐angle recognition results obtained by numerical calculation, FDTD full‐wave simulation, and experimental measurement. (a–c) Output intensity distributions for an incident state of (−3.9°,−3.9°) obtained by calculation (Cal.), simulation (Sim.), and experiment (Exp.). (d–f) Integrated detector‐energy distributions corresponding to panels (a–c). The α and β detector groups are normalized separately, and blue bars indicate the correct detectors.

To comprehensively evaluate the classification performance of the DA‐DNN system, we generated the confusion matrices shown in Figure 6 from extensive data obtained by calculation and experiment. The panels present the calculated α and β matrices followed by their experimental counterparts. In all confusion matrices, the responses are concentrated mainly along the principal diagonal, clearly indicating an accurate mapping between the true angular intervals and the detector regions predicted by the network and thus demonstrating the high classification accuracy of the system. Off‐diagonal experimental responses arise mainly from inherent crosstalk between adjacent detector regions caused by diffraction, as well as experimental noise. These effects may be further reduced by optimizing the interlayer distances or using detector arrays with higher spatial resolution.

**FIGURE 6:**
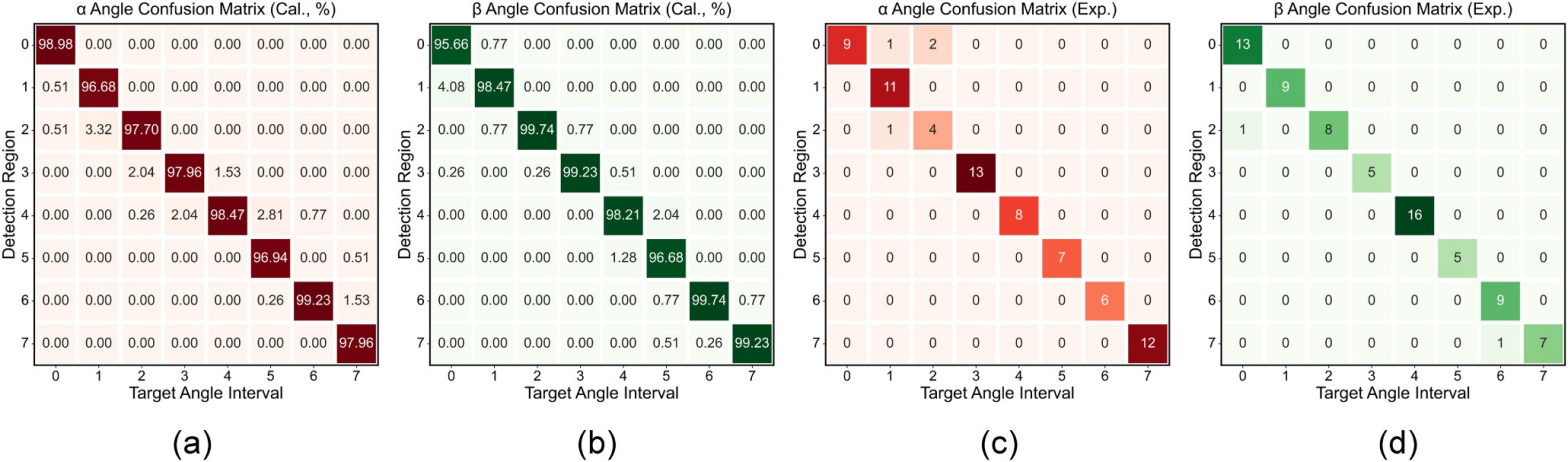
Confusion matrices for α and β classification obtained by numerical calculation and experimental measurement. The horizontal axis denotes the true angular class, the vertical axis denotes the predicted detector class, and the color intensity represents the percentage or number of samples assigned to each class.

Overall, the high agreement between the equivalent dual‐angle experiments and full‐wave simulations strongly demonstrates that the proposed DA‐DNN architecture can reliably distinguish and recognize multiple incident angles with high accuracy. These results reveal the feasibility and substantial potential of physically interpretable DNNs for DoA sensing at multiple angles in the THz band. The demonstrated performance is consistent with our overall objective of developing a passive and scalable optical DoA estimation framework with low latency for future wireless communication systems. UAV communication is one potential application of such a system. The present design and experiments address one incident signal. Extending the system to simultaneous signals would require a multilabel optical readout and a mechanism for pairing the two angular components associated with each source. Time‐resolved or polarization‐resolved measurements combined with electronic pairing may provide one route to this extension. This work establishes a rigorous experimental foundation for the further development and practical deployment of this technology.

## 3 Conclusion

In conclusion, we solve the fundamental problem of passive and simultaneous 2D DoA recognition using a single diffractive device, overcoming the dependence of previous 1D methods on mechanical rotation. Through theoretical analysis and experimental validation, we demonstrate that the proposed spatial encoder–decoder architecture can accurately resolve the complete 2D angular information of incident light. In this architecture, the spatial encoder, implemented by a cross‐shaped diffractive aperture, converts the phase distribution of the incident wavefront into a richly structured spatial intensity pattern. This transformation significantly enhances the amplitude contrast between signals arriving from different directions, enabling the subsequent diffractive layers to robustly decode both angular dimensions within a single integrated network.

We fabricated a compact DA‐DNN device using 3D printing at high resolution and validated its performance through extensive numerical calculations, full‐wave FDTD modeling, and THz experiments. The results confirm that the device performs reliable and accurate direction estimation over a wide FOV covering 64 distinct 2D angular combinations. This work therefore establishes an experimentally validated architecture for efficient simultaneous 2D angular detection using a passive and physically interpretable optical system. Future work will further optimize the architecture through systematic ablation studies of the network parameters and explore advanced materials with low optical loss, such as ceramics, to support deeper and more complex diffractive networks. Overall, this study provides a pathway toward compact all‐optical front ends with low latency and high energy efficiency for future THz communication platforms operating in real time.

## 4 Experimental Section

### 4.1 Training

The DA‐DNN was trained using Python 3.12.11 and PyTorch 2.5.1 on a Windows 10 workstation equipped with an Intel Xeon W‐2275 3.30 GHz processor, 128 GB of memory, and an NVIDIA GeForce RTX 3090 graphics processor. Within each DOE layer, the trained HCFR‐Net calculated the output complex field from the incident complex field and quantized height map. Propagation between finite planes was calculated using the Rayleigh–Sommerfeld diffraction integral.

### 4.2 Experimental Setup

A custom optical setup was constructed to validate the device performance. A 0.3‐THz continuous‐wave IMPATT diode (TeraSense) was used as the coherent source. The emitted beam was collimated and expanded by two TPX lenses (Tydex), producing a uniform plane wave that covered the device aperture. After transmission through the DA‐DNN, a fast linear array detector (TeraFAST, TeraSense) recorded the transmitted intensity distribution through 2D scanning. The measured intensity data were subsequently processed to obtain the angular predictions.

## Author Contributions

**Chenjie Xiong:** data curation, software, methodology, conceptualization, formal analysis, visualization, writing – original draft, writing – review and editing, investigation. **Shuo Wang:** software, validation. **Jianzhou Huang:** software, investigation. **Jia Zhang:** resources, project administration. **Luosha Zhang:** software, project administration. **Yu Wang:** supervision. **Jin Tao:** supervision, funding acquisition. **Chengjun Zou:** writing – review and editing, supervision, project administration, conceptualization, funding acquisition. **Bin Hu:** project administration, supervision, writing – review and editing, conceptualization, funding acquisition.

## Conflicts of Interest

The authors declare no conflicts of interest.

## Supporting information


Supporting Information S1

**Video S1:** Experimental dual‐angle detection for equivalent incident angles (*α*, *β*) = (−3.9°,−3.9°). The video shows the scanning measurement of the output intensity distribution of the dual‐angle diffractive neural network (DA‐DNN) under 0.3 THz continuous wave illumination. The measured pattern is displayed on the monitor. The magenta and yellow annotations in the final frame identify the dominant detector regions *α*3 and *β*3, respectively. The corresponding experimental intensity map and detector‐energy distributions are presented in Figure 5c,f of the main text.

**Video S2:** Experimental dual‐angle detection for equivalent incident angles (*α*, *β*) = (−12.8°,−12.8°). The video records the scanning measurement of the DA‐DNN output intensity distribution under 0.3 THz continuous wave illumination, with the measured pattern displayed on the monitor. The corresponding detector‐energy distributions peak at detector 2 in both the *α* and *β* groups, allowing the two angular components to be read from the same output pattern. This measurement corresponds to the top row of Figure S6 in Section S7 of the Supporting Information.

## Data Availability

The data that support the findings of this study are available in the Supporting Information of this article.
