# Supplementary material for: All‐Optical Dual‐Angle Diffractive Neural Networks for 2D Direction‐of‐Arrival Estimation
Source: Nanophotonics. 2026 Oct 22;15(20):e70315. doi: 10.1002/nap2.70315 (PMC13687936; doi:10.1002/nap2.70315)
Supplement: Supplementary file 1 [file NAP2-15-e70315-s003.pdf]

Supporting Information

All-optical Dual-Angle Diffractive Neural Network for Two-Dimensional Direction-of-Arrival Estimation

Chenjie Xiong[1,2], Shuo Wang[3,4], Jianzhou Huang[1,2], Jia Zhang[1,5], Luosha Zhang[6], Yu Wang[3], Jin Tao[7], Chengjun Zou[3], Bin Hu[1,2]

[1]School of Optics and Photonics, Beijing Institute of Technology, Beijing 100081, China
[2]National Key Laboratory on Near-surface Detection, Beijing 100072, China
[3]Institute of Microelectronics, Chinese Academy of Sciences, 3 Beitucheng West Road, Beijing 100029, China
[4]University of Chinese Academy of Sciences, Beijing 101408, China
[5]China Academy of Aerospace Science and Innovation, Beijing 100089, China
[6]Institute of Applied Physics, Abbe Center of Photonics, Friedrich Schiller University Jena, Jena 07745, Germany
[7]State Key Laboratory of Optical Communication Technologies and Networks, China Information and Communication Technologies Group Corporation, Wuhan 430205, China

Contents

- **S1.** Angular Definitions, Class Binning, and Detector Allocation
- **S2.** HCFR-Net Complex Field Mapping
- **S3.** Ablation of Aperture Shape
- **S4.** Ablation of Cross Aperture Size
- **S5.** Equivalent Setup and Device Photographs
- **S6.** Numerical and FDTD Comparison
- **S7.** Additional Experimental Results
- Supplementary Video Captions

S1 Angular Definitions, Class Binning, and Detector Allocation

This section defines a common mapping between the incident direction, discrete angle classes, and output detectors. The same definitions are used for training labels, numerical calculations, FDTD simulations, and experimental readout.

Let $\mathbf{s} = (s_x, s_y, s_z)$ denote the direction from the device origin to the target, with $s_z > 0$. The incident unit wavevector $\hat{\mathbf{k}}_{\text{in}}$ is antiparallel to $\mathbf{s}$. The conventional zenith angle θ is measured from the positive z-axis. The azimuth angle φ is measured in the xy-plane from the positive x-axis toward the positive y-axis. We define α as the signed angle between the xz-plane projection of $\mathbf{s}$ and the positive z-axis, with rotation toward positive x taken as positive. Similarly, β is defined using the yz-plane projection, with rotation toward positive y taken as positive. Thus,

$$\tan\alpha = \frac{s_x}{s_z} = \tan\theta\cos\varphi, \qquad \tan\beta = \frac{s_y}{s_z} = \tan\theta\sin\varphi. \tag{S1}$$

The inverse transformation is

$$\theta = \tan^{-1}\left(\sqrt{\tan^2\alpha + \tan^2\beta}\right), \qquad \varphi = \operatorname{atan2}(\tan\beta, \tan\alpha). \tag{S2}$$

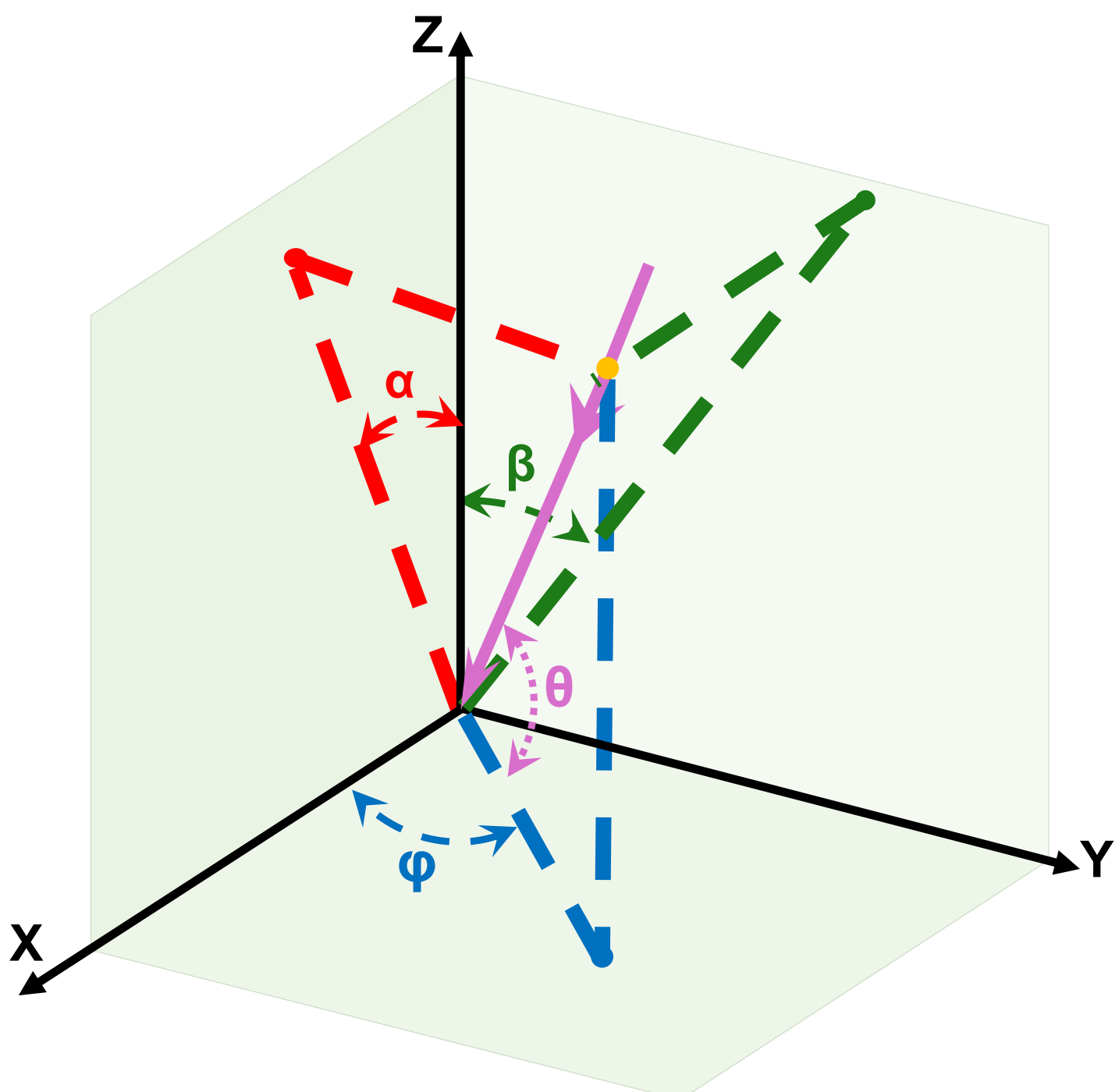

Fig. S1. Definition of the angular coordinates. The vector $\mathbf{s}$ points from the device origin toward the target, and $\hat{\mathbf{k}}_{\text{in}}$ is antiparallel to $\mathbf{s}$. The red and green dashed lines show the projections of $\mathbf{s}$ onto the xz- and yz-planes, respectively. The angles α and β are positive from $+z$ toward $+x$ and $+y$, respectively. The blue dashed line shows the projection onto the xy-plane. The azimuth angle φ is positive from $+x$ toward $+y$, and θ is the angle between $\mathbf{s}$ and the positive z-axis.

Both α and β span $[-40°, 40°)$ and are divided into eight 10° intervals that are closed on the left and open on the right. Table S1 uses the 60×60 output array coordinates in Fig. 1c of the main text. The coordinate x_{pix} increases from left to right, and y_{pix} increases from top to bottom. Each detector region covers 4×4 pixels.

Table S1. Angle classes and spatially separated $8 + 8$ detector layout. Classes 0–7 correspond to eight 10° intervals that are closed on the left and open on the right for both α and β. The final two columns give the detector center coordinates on the 60×60 output array. Each detector region covers 4×4 pixels.

Class	Angular interval	α detector $(x_{\text{pix}}, y_{\text{pix}})$	β detector $(x_{\text{pix}}, y_{\text{pix}})$
0	$[-40^\circ, -30^\circ)$	$(50, 20)$	$(20, 10)$
1	$[-30^\circ, -20^\circ)$	$(50, 30)$	$(30, 10)$
2	$[-20^\circ, -10^\circ)$	$(50, 40)$	$(40, 10)$
3	$[-10^\circ, 0^\circ)$	$(40, 30)$	$(30, 20)$
4	$[0^\circ, 10^\circ)$	$(20, 30)$	$(30, 40)$
5	$[10^\circ, 20^\circ)$	$(10, 20)$	$(20, 50)$
6	$[20^\circ, 30^\circ)$	$(10, 30)$	$(30, 50)$
7	$[30^\circ, 40^\circ)$	$(10, 40)$	$(40, 50)$

Each incident state therefore maps to two discrete classes that are read independently by two spatially separated detector groups. All subsequent sections use the same class intervals and detector coordinates.

S2 HCFR-Net Complex Field Mapping

This section defines the inputs and outputs of the Height-Conditioned Complex-Field Response Network (HCFR-Net) and summarizes the complex field response data used for its training and evaluation. HCFR-Net follows the response modeling concept of KBNN introduced by Ma *et al.*[1]. KBNN maps an ideal phase distribution to the effective phase obtained after accounting for electromagnetic coupling between adjacent elements. Based on this concept, HCFR-Net calculates the transmitted complex vector field from the incident complex vector field and the DOE height map. The mapping represents field response variations caused by oblique incidence and local electromagnetic coupling between adjacent elements.

For DOE layer l, HCFR-Net receives the incident complex vector field $\mathbf{E}_l^{\text{in}}$ and the corresponding height map $\mathbf{H}_l$ quantized into eight levels. It calculates the transmitted complex vector field $\mathbf{E}_l^{\text{out}}$ immediately after the DOE layer:

$$\mathbf{E}_l^{\text{out}} = \text{HCFR-Net}\big(\mathbf{E}_l^{\text{in}}, \mathbf{H}_l\big)\,, \qquad l = 1, 2. \tag{S3}$$

Both $\mathbf{E}_l^{\text{in}}$ and $\mathbf{E}_l^{\text{out}}$ contain the complex E_x, E_y, and E_z field components. The mapping therefore retains the amplitude, phase, and vector components of the electromagnetic field. Within the optical propagation model of the DA-DNN, HCFR-Net calculates the field transformation through each DOE layer. The α and β detector arrays on the output plane subsequently read the two angular classes.

The data used to train and evaluate HCFR-Net were obtained from FDTD simulations. The dataset covers 181 distinct height maps quantized into eight levels. These height maps were combined with different incidence conditions whose angular coordinates range from -40° to 40°. The corresponding incident and transmitted complex vector fields form 586 paired field responses for individual DOE layers. Of these 586 field pairs, 427 were used for training, 63 for validation, and 96 for testing.

The dataset contains DOE regions with sizes of 24×24, 48×48, and 60×60 pixels. These dimensions denote the numbers of DOE pixels along the two transverse directions. The three region

sizes were selected to balance the fidelity with which local electromagnetic coupling is represented against the computational cost of FDTD data generation. The 24×24 pixel regions allow a broader range of DOE height maps to be sampled at lower computational cost, whereas the 48×48 and 60×60 pixel regions retain larger surrounding electromagnetic environments for selected height maps and incidence conditions. The three sizes contain 195, 157, and 234 field response pairs and cover 131, 16, and 34 distinct height maps, respectively. Within the training set, the three sizes contain 124, 143, and 160 field pairs.

Different incidence conditions are supplied to HCFR-Net through their corresponding incident complex vector fields $\mathbf{E}_l^{\text{in}}$. Their effects on the field amplitude and phase are encoded directly in the input complex field.

S3 Ablation of the Aperture Shape

This section examines the role of the input aperture in spatial encoding. We compare classification accuracy and the energy fraction in the target detector across aperture shapes under otherwise matched task settings.

The ablation includes seven apertures and a no aperture input, giving eight conditions in total. Let $M(x, y)$ denote the aperture amplitude transmission function. The field immediately after the aperture is

$$E_A(x, y) = M(x, y) \odot E_{\text{inc}}(x, y). \tag{S4}$$

Here, $\odot$ denotes element-wise multiplication on the sampled transverse plane.

Propagation to E_1^{in} then produces amplitude and phase distributions that vary with angle. The four angle ranges are centered at zero and span 80°, 40°, 16°, and 8°. Each range contains eight classes, with class widths of 10°, 5°, 2°, and 1°, respectively. The α and β accuracies measure the two components separately. Joint accuracy requires both components to be correct. The target detector energy fraction is the energy in the target region divided by the total energy in the corresponding detector group of eight regions.

Figure S2 compares six aperture shapes with equal areas, a $50\,\text{px} \times 10\,\text{px}$ cross aperture, and the no aperture input. Across the 80° range, the cross aperture achieved 96.36% joint accuracy. The no aperture input achieved 87.65%, while the other six shapes achieved 92.65%–95.43%. For the 40° range, the cross and no aperture conditions achieved 99.17% and 98.72%, respectively. Their corresponding accuracies were 97.80% and 99.66% over 16°, and 96.60% and 98.95% over 8°. The benefit of the cross aperture was most pronounced across the widest tested range.

The role of the aperture shape therefore depends on the angular range. The $50\,\text{px} \times 10\,\text{px}$ cross aperture performed best on the 80° primary task. Its orthogonal arms also provide spatial encoding aligned with the α and β readouts. Over narrower ranges, the no aperture input or another shape can yield comparable or higher accuracy. The selected shape is therefore specific to the task covering the widest angular range and is not universally optimal.

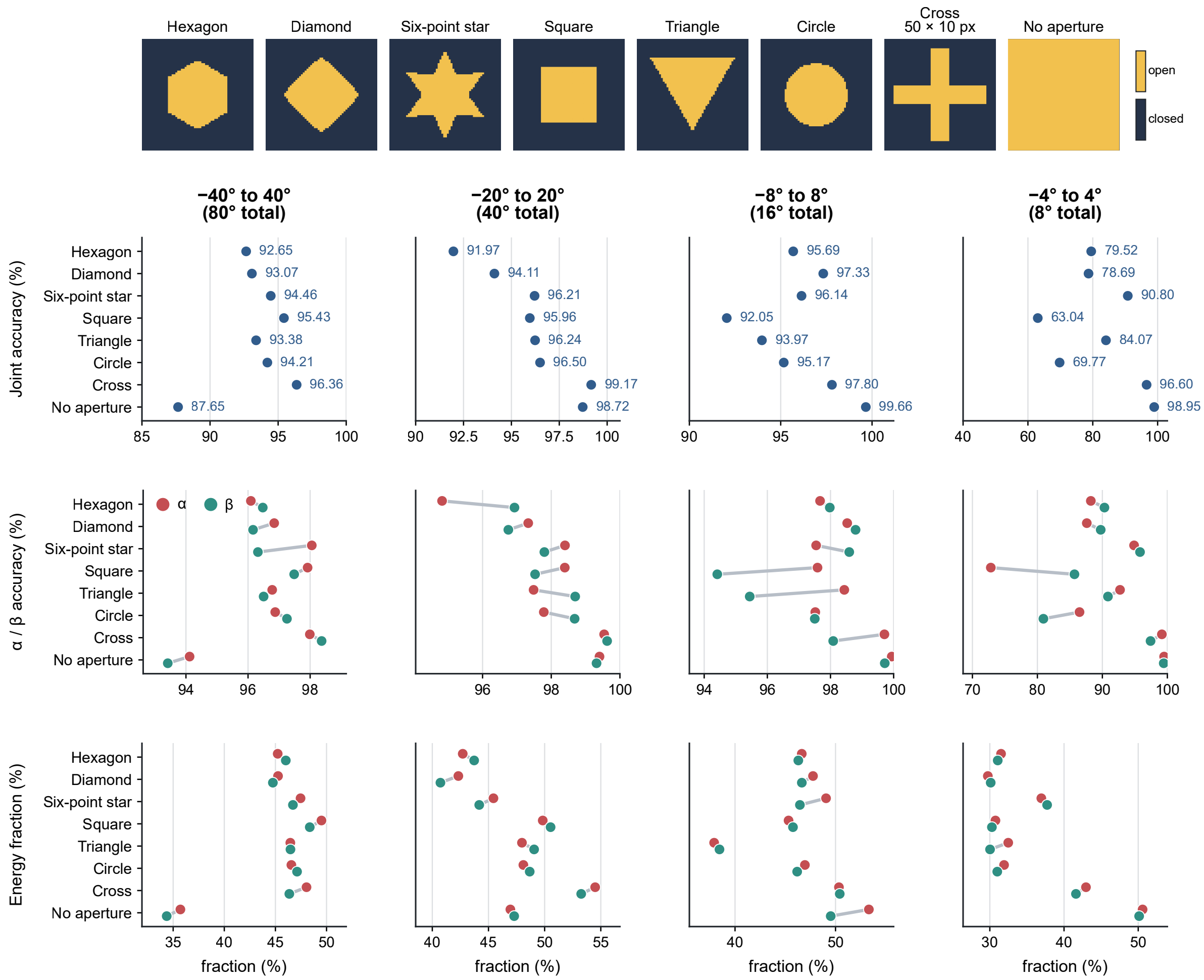

Fig. S2. Ablation of aperture shape over four angular ranges. The top row shows six openings with equal areas, a 50 px × 10 px cross aperture, and the no aperture input. Yellow and dark regions denote open and blocked areas, respectively. The four columns correspond to total angular spans of 80°, 40°, 16°, and 8°, each divided into eight classes. The next three rows report joint accuracy, α/β accuracy, and target detector energy fraction. Red and green markers denote α and β, respectively. Gray lines connect paired results for the same aperture condition.

S4 Ablation of the cross Aperture Size

After selecting the cross opening, we examined how arm length and width affect spatial encoding. Five aperture sizes were evaluated using the same four angular ranges and three metrics as in Fig. S2. This design isolates aperture geometry as the comparison variable.

Figure S3 compares the five cross apertures. The 50 px × 10 px design achieved the highest joint accuracy over the 80° and 40° ranges, reaching 96.36% and 99.17%, respectively. Over the 16° range, the 50 px × 30 px aperture reached 99.08%, while the 50 px × 10 px aperture reached 97.80%. Over the 8° range, the 50 px × 10 px aperture again achieved the highest joint accuracy at 96.60%. In comparison, the 30 px × 10 px aperture achieved 62.43%.

The optimal size changes across angular ranges. Target detector energy fraction and joint

accuracy also do not follow a simple monotonic relationship. Neither optical throughput nor energy concentration alone therefore predicts the classification performance for both angular components. We selected the 50 px × 10 px aperture because it maximized joint accuracy on the 80° primary task while retaining a measurable output signal.

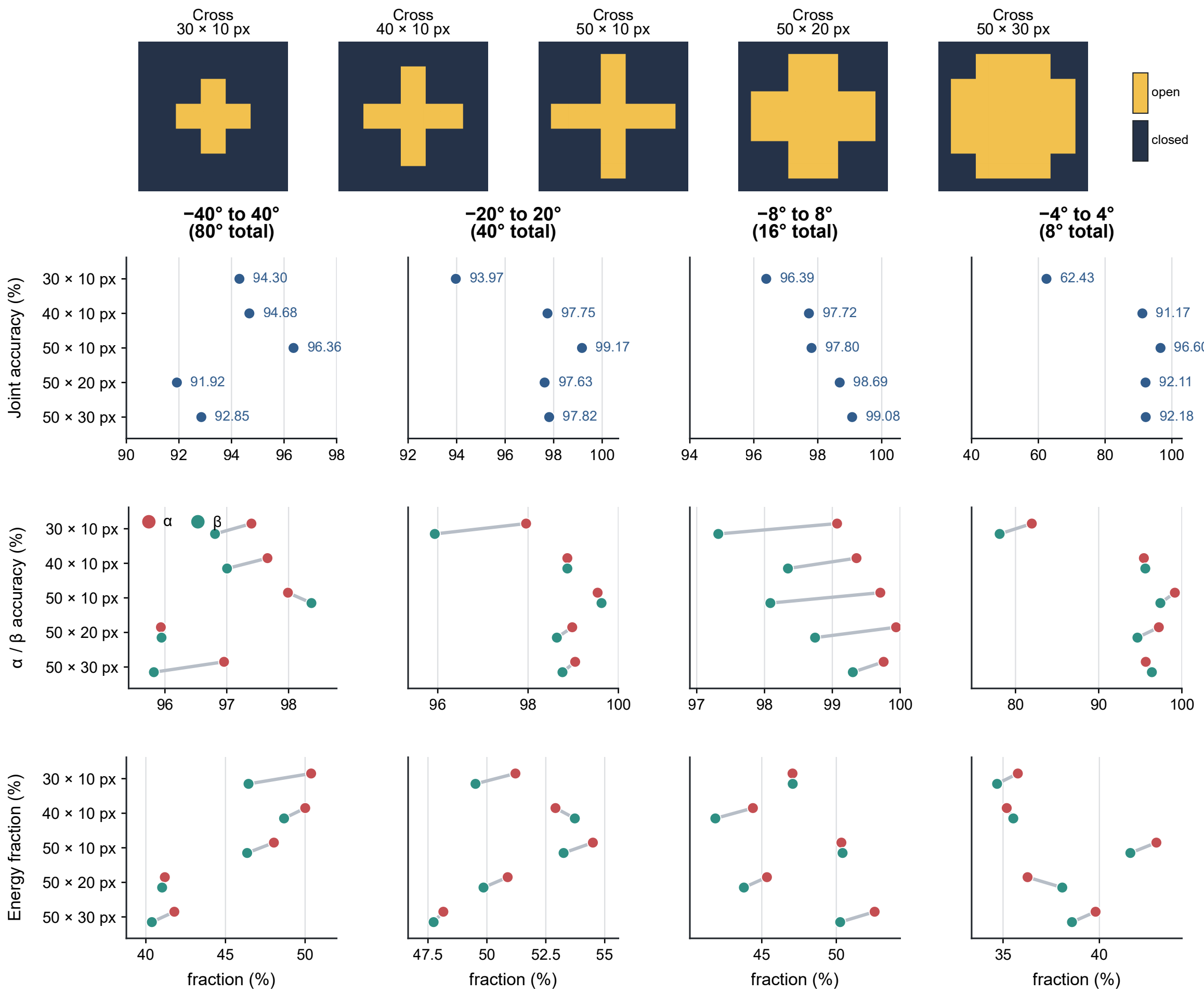

Fig. S3. Ablation of cross aperture size over four angular ranges. The top row shows openings of 30 px × 10 px, 40 px × 10 px, 50 px × 10 px, 50 px × 20 px, and 50 px × 30 px. Yellow and dark regions denote open and blocked areas, respectively. The four columns correspond to total angular spans of 80°, 40°, 16°, and 8°. The next three rows report joint accuracy, α/β accuracy, and target detector energy fraction. Red and green markers denote α and β, respectively. Gray lines connect paired results for the same aperture size.

S5 Equivalent Setup and Device Photographs

This section documents the equivalent geometry for measuring two angular components and the fabricated device. The device and detector are rotated together to change the zenith direction. The device is also rotated about its center to construct different equivalent states defined by two angular components. Front and rear photographs record the packaged input surface, cross aperture, and mounting orientation.

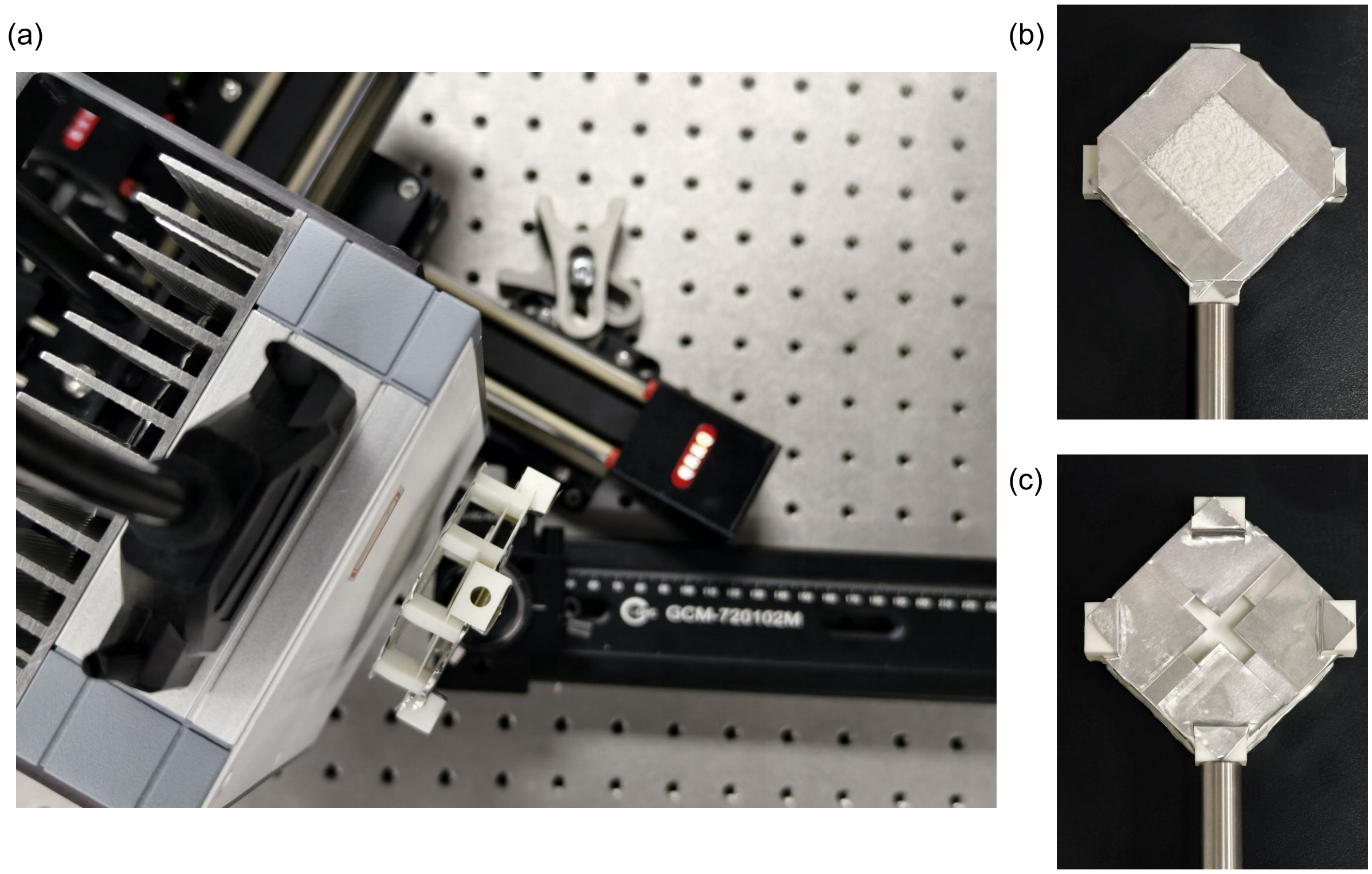

Fig. S4. Equivalent experimental setup for measuring two angular components and the fabricated device. a, Photograph of the aligned setup. The device and detector are rotated together through θ, and the device can be set to azimuthal states of $\varphi = 45°$, $135°$, $225°$, and $315°$ about its center. b, Front view of the fabricated device, showing the packaged input surface. c, Rear view, showing the cross aperture and its position relative to the device frame.

The photographs establish the physical geometry of the experiment based on equivalent rotation and distinguish the two device faces. They connect the mounting orientation to the angular definitions and experimental results in the main text.

S6 Numerical and FDTD Comparison

This section compares the numerical propagation model with FDTD simulations for six representative inputs defined by two angular components. Both methods use the same DOE, detector regions, and normalization within each detector group. We compare the output intensity and the energy percentages across all 16 detector regions.

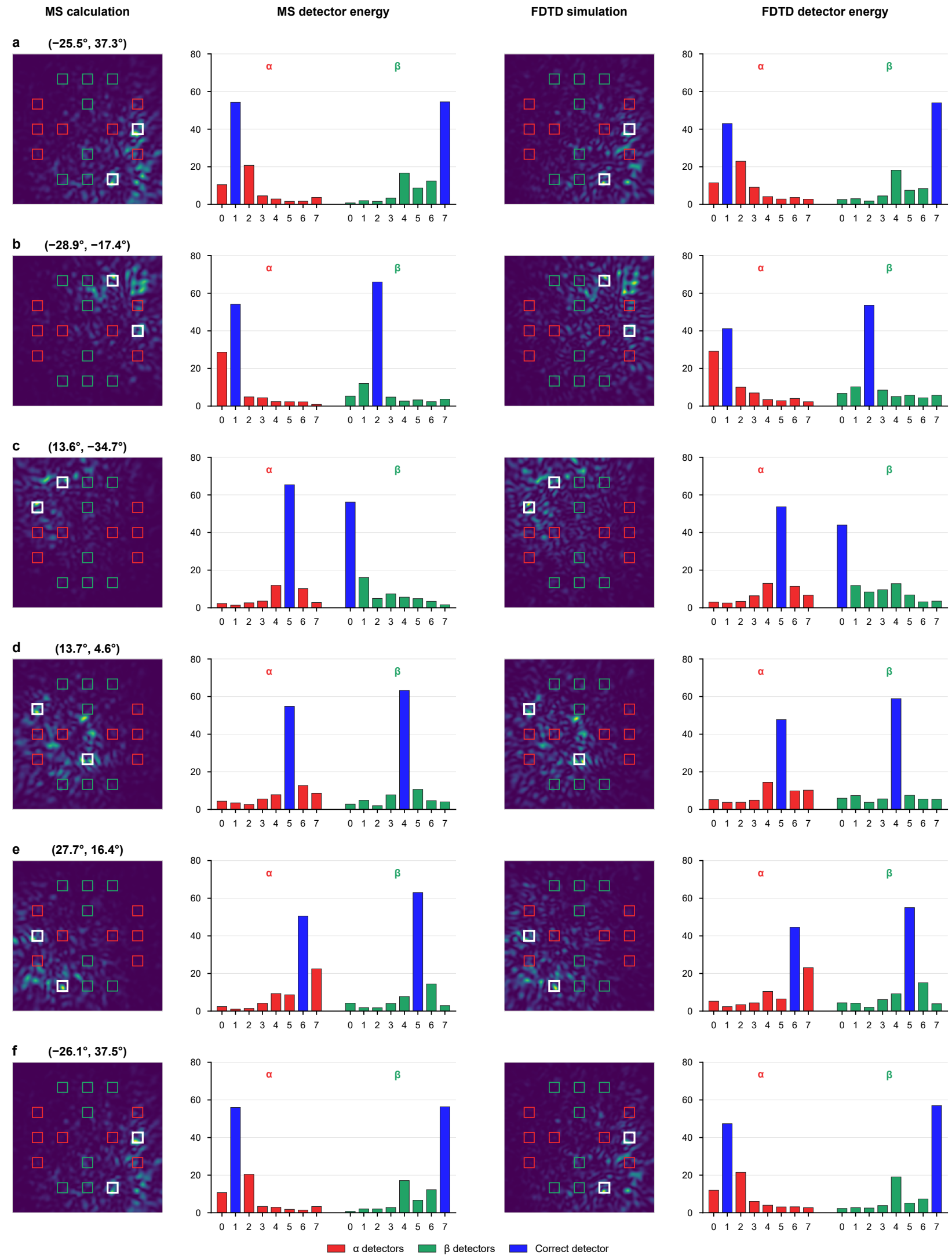

Fig. S5. Comparison between numerical calculations and segmented FDTD simulations for six representative inputs defined by two angular components. Panels a–f correspond to $(-25.5°, 37.3°)$, $(-28.9°, -17.4°)$, $(13.6°, -34.7°)$, $(13.7°, 4.6°)$, $(27.7°, 16.4°)$, and $(-26.1°, 37.5°)$, respectively. The four columns in each row show the numerically calculated intensity, calculated detector energy, FDTD intensity, and FDTD detector energy. Red and green boxes or bars denote the α and β detector groups. White outlines and blue bars identify the target detectors for the corresponding angles. The two detector groups are normalized separately over their eight regions.

Both methods direct the dominant α and β energies to the same target classes in all six cases. Local intensity textures and energy distributions outside the target detectors differ between the two methods without changing these class assignments. The comparison supports consistent detector assignments for the selected inputs. Because it includes six representative cases, it does not replace an accuracy assessment over the full angular range.

S7 Additional Experimental Results

This section provides three additional measurements of two angular components. These cases test whether the device maps both angular components to their target regions across different quadrants and angle magnitudes. The incident angle pairs are $(-12.8°, -12.8°)$, $(25.9°, -25.9°)$, and $(35.1°, -35.1°)$. The corresponding measurement of $(-12.8°, -12.8°)$ is provided in Video S2.

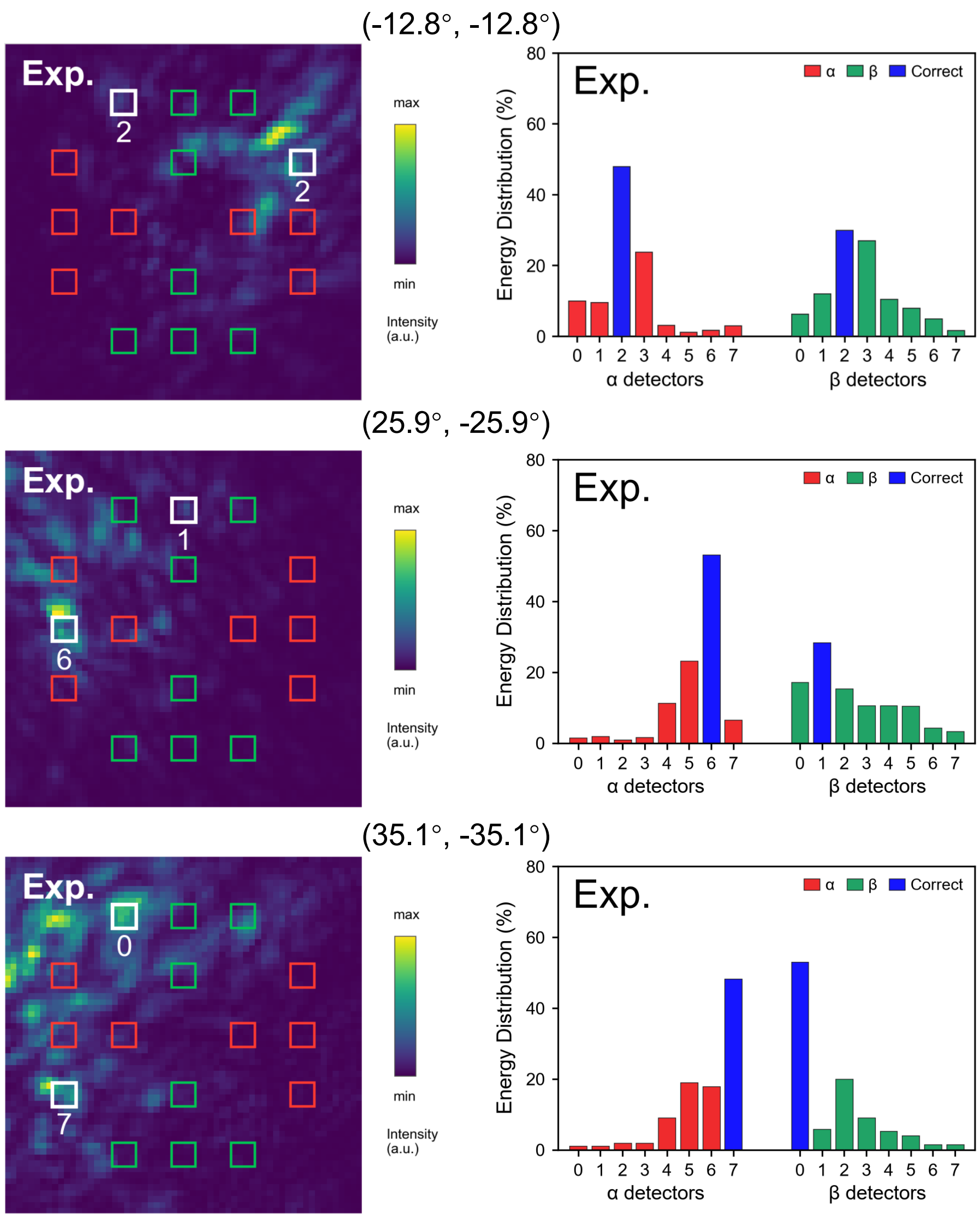

Fig. S6. Experimental output intensity and detector energy distributions for three inputs defined by two angular components. The top, middle, and bottom rows correspond to $(-12.8°, -12.8°)$, $(25.9°, -25.9°)$, and $(35.1°, -35.1°)$, respectively. In each row, the left panel shows the measured output intensity, and the right panel shows the corresponding detector energy fractions in the α and β groups. Red and green boxes mark the α and β detector regions in the intensity maps. White boxes and adjacent numbers identify the target detectors and their indices. Red and green bars denote the detector energy fractions in the α and β groups, respectively, and blue bars highlight the target detectors. The two detector groups are normalized separately over their eight regions.

For all three measurements, the dominant energy occurs in the corresponding white target regions. The two angular components can therefore be read simultaneously for these measured states. These results extend the representative experiments in the main text but do not replace accuracy statistics over the full angular range.

Supplementary Video Captions

Video S1. Experimental dual-angle detection for equivalent incident angles $(\alpha, \beta) = (-3.9°, -3.9°)$**.** The video shows the scanning measurement of the output intensity distribution of the dual-angle diffractive neural network (DA-DNN) under 0.3 THz continuous-wave illumination. The measured pattern is displayed on the monitor. The magenta and yellow annotations in the final frame identify the dominant detector regions $\alpha 3$ and $\beta 3$, respectively. The corresponding experimental intensity map and detector-energy distributions are presented in Figure 5c,f of the main text.

Video S2. Experimental dual-angle detection for equivalent incident angles $(\alpha, \beta) = (-12.8°, -12.8°)$**.** The video records the scanning measurement of the DA-DNN output intensity distribution under 0.3 THz continuous-wave illumination, with the measured pattern displayed on the monitor. The corresponding detector-energy distributions peak at detector 2 in both the α and β groups, allowing the two angular components to be read from the same output pattern. This measurement corresponds to the top row of Figure S6 in Section S7 of the Supporting Information.

References

[1] Ma, Z. *et al.* High-fidelity terahertz diffractive neural networks via KBNN-integrated design. *Laser Photonics Rev.* **20**, e02611 (2026). https://doi.org/10.1002/lpor.202502611.
